# Central Compartment Lymph Nodes Have Distinct Metastatic Patterns in Different Age Groups

**DOI:** 10.3389/fendo.2022.807431

**Published:** 2022-02-17

**Authors:** Caigu Yan, Xianghui He, Zuoyu Chen, Yizeng Wang

**Affiliations:** ^1^ Department of General Surgery, The People’s Hospital of Liuyang, Changsha, China; ^2^ Department of General Surgery, Tianjin Medical University General Hospital, Tianjin, China

**Keywords:** papillary thyroid carcinoma, central compartment lymph nodes metastasis, age, thyroglobulin, Hashimoto’s thyroiditis, prognosis

## Abstract

**Background and Purpose:**

Central compartment lymph node metastasis (CLNM) is a manifestation of tumor aggressiveness and an indicator of tumor prognosis. The purpose of this study was to construct a nomogram for evaluating CLNM patterns in papillary thyroid carcinoma (PTC) in different age groups.

**Method:**

A total of 907 patients diagnosed with PTC from August 2014 to December 2018 were enrolled. A nomogram illustrating CLNM was generated using the results of multivariate logistic regression analysis.

**Results:**

According to the best Youden index, we set the cut-off age at 45 years. Multivariate logistic regression analysis showed that in patients aged <45 years, large tumor size (P<0.05), extra-thyroid extension (P<0.05) and thyroglobulin level >40 ng/ml (OR=2.985, 95% CI 1.379-6.462; P<0.05) were independent risk factors; meanwhile, Hashimoto’s thyroiditis (OR=0.532, 95% CI 0.324-0.874; P<0.05) was a protective factor of CLNM. In the subgroup with age ≥45 years, large tumor size (P<0.05), extra-thyroid extension (P<0.05), unclear margin (OR=1.604, 95% CI 1.065-2.416; P<0.05), male gender (OR=2.009, 95% CI 1.257-3.212; P<0.05) were independent risk factors for CLNM. In the subgroup with age <45 years, an area under the curve (AUC) of 0.729 (95% CI 0.680-0.777); P<0.05) was obtained. In the ≥45 years subgroup, the AUC was 0.668 (95% CI 0.619-0.716; P<0.05).

**Conclusion:**

CLNM of PTC in different age groups may have distinct patterns. Based on the potential risk factors for CLNM in patients with different age stratification, a user-friendly predictive model was established.

## Background

Thyroid cancer is becoming one of the most common malignant human tumors. The incidence of thyroid cancer has continued to rise around the world in the past few decades (1, 2). Studies have shown that papillary thyroid carcinoma (PTC) has high rates of central compartment lymph nodes metastasis (CLNM), ranging from 20% to 90% (3). Prophylactic central lymph node dissection remains controversial (4–6). Some investigators believe that micrometastasis to lymph nodes does not affect the survival time of patients, while other studies have pointed out that prophylactic lymph node dissection could increase the recurrence free-survival rate and reduce postoperative serum thyroglobulin levels, yielding accurate staging to assist further treatment. Moreover, due to the use of nerve monitoring and carbon nanoparticle negative imaging (7, 8), permanent damage to the recurrent laryngeal nerve and parathyroid gland is rarely found during dissection of the initial central compartment lymph nodes. Because PTC has a good postoperative survival time, more attention is paid to the probability of postoperative recurrence. when PTC is recurrent and reoperated, it is more likely to cause permanent damage to the recurrent laryngeal nerve and parathyroid gland due to changes in anatomical structure.

CLNM is a manifestation of tumor aggressiveness and an indicator of tumor prognosis. There are many studies assessing the risk factors for CLNM (9–12). However, studies assessing CLNM of thyroid cancer are based on the overall population without age stratification. It is well known that age is a significant factor affecting the biological behavior of thyroid cancer (13). The 8^th^ edition of American Joint Committee on Cancer guidelines indicate elderly patients have decreased disease-free survival (14). Due to different hormone levels and immune states in patients of different ages (15–18), distinct patterns of CLNM may be present.

The purpose of this study was to explore central compartment lymph nodes that have distinct metastatic patterns in different age groups and to assist in clinical decision-making. At the same time, we examined the potential differences in the biological behavior of thyroid cancer in different ages.

## Materials and Methods

### Clinical Data

**Figure 1:**
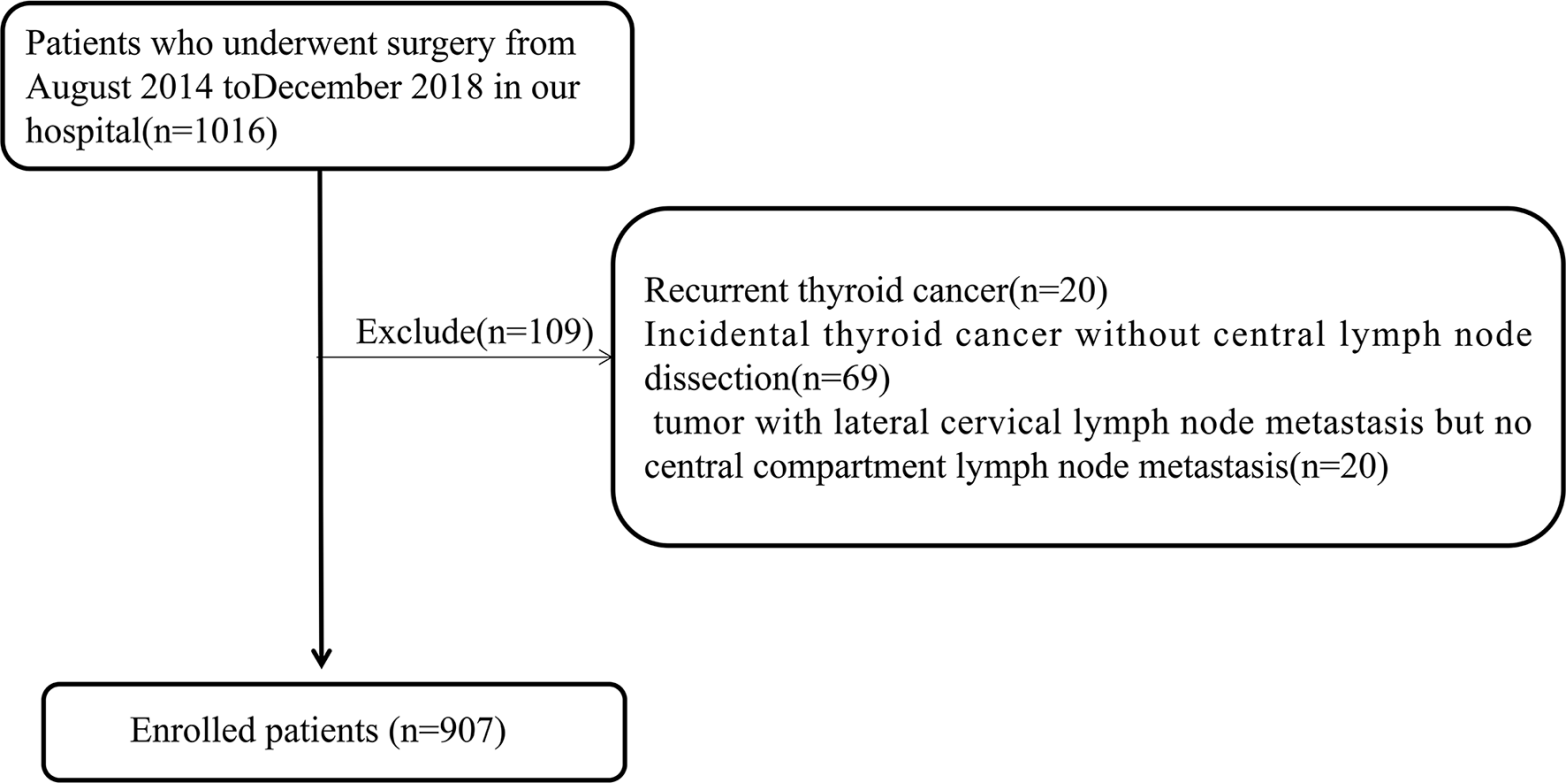
The flow chart of the patients enrolled in this study.

This was a retrospective analysis. A total of 907 patients diagnosed with papillary thyroid carcinoma admitted to a tertiary hospital from August 2014 to December 2018 and operated by the same surgeon were selected as subjects. This study passed the ethical review based on the Declaration of Helsinki, and obtained informed consent from the patients. Preoperatively, all patients underwent fine-needle aspiration cytology (FNA) guided by ultrasound to determine the pathological features, and BRAF V600E was detected at the same time for further diagnosis. Inclusion criteria were: 1) initial thyroid cancer surgery; 2) postoperative pathology confirming thyroid papillary carcinoma; 3) at least unilateral lobectomy plus regional lymph node dissection; 4) completed clinical and pathological data. Exclusion criteria were: 1) recurrent thyroid cancer; 2) incidental thyroid cancer without central lymph node dissection; 3) tumor with lateral cervical lymph node metastasis but no central compartment lymph node metastasis. **Figure 1** shows the flow chart of the patients enrolled in this study.

**Figure 2:**
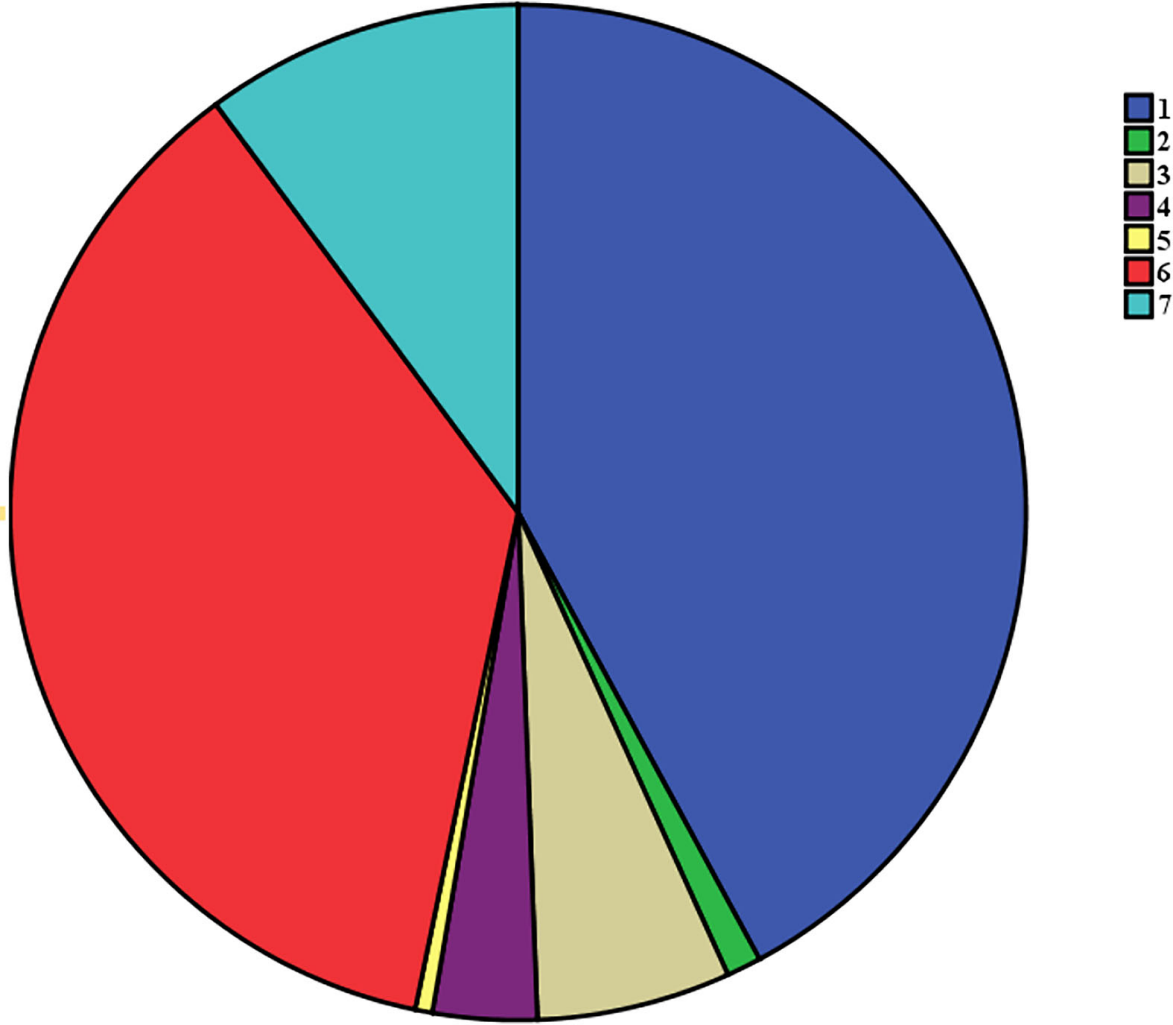
The flow chart of all different surgical procedures:1) ipsilateral glandular lobe + isthmus resection + unilateral central compartment lymph node dissection; 2) ipsilateral glandular lobe + isthmus resection + unilateral central compartment lymph node dissection + lateral neck area dissection; 3)total thyroidectomy + unilateral central compartment lymph node dissection + lateral neck area dissection; 4) total thyroidectomy + bilateral central compartment lymph node dissection + unilateral neck area dissection; 5) total thyroidectomy + bilateral central compartment lymph nodes dissection + bilateral neck area dissection; 6) total thyroidectomy + unilateral central compartment lymph nodes dissection; 7) total thyroidectomy + bilateral central compartment lymph nodes dissection.

All 907 patients underwent standardized surgical treatment, all operations were traditional surgeries rather than endoscopic surgeries. In our center, all patients diagnosed with thyroid papillary carcinoma underwent routine prophylactic central compartment lymph node dissection at least on one side. The surgical methods were as follows: 1) ipsilateral glandular lobe + isthmus resection + unilateral central compartment lymph node dissection (382 cases); 2) ipsilateral glandular lobe + isthmus resection + unilateral central compartment lymph node dissection + lateral neck area dissection (10 cases); 3) total thyroidectomy + unilateral central compartment lymph node dissection + lateral neck area dissection (56 cases); 4) total thyroidectomy + bilateral central compartment lymph node dissection + unilateral neck area dissection (30 cases); 5) total thyroidectomy + bilateral central compartment lymph nodes dissection + bilateral neck area dissection (5 cases); 6) total thyroidectomy + unilateral central compartment lymph nodes dissection (332 cases); 7) total thyroidectomy + bilateral central compartment lymph nodes dissection (92 cases). All surgical methods are shows in **Figure 2**.

We set the cut-off age at 45 years, which is in line with the age stratification of the 7^th^ edition of the AJCC, and obtained the best Youden index (low false-positive rate or/and high sensitivity). Clinical data included general patient features (gender, age, chief complaint and history of surgery), laboratory data (TSH, FT3, FT4, CEA, Tg, Tg-Ab, anti-TPO and PTH), ultrasonographic findings, fine-needle aspiration results, postoperative pathology, length of hospital stay, operation time, intraoperative frozen pathology and final pathological diagnosis. Clinical features were defined as follows: 1)pre-surgical data: gender (male or female), duration of chief complaint (less than 1 year or more than 1 year), aspect ratio (height divided by width on transverse views, A/T), margin on ultrasonogram (clear or unclear), calcification on ultrasonogram (absent or present), TSH (less than 2.7 uIU/ml or more than 2.7 uIU/ml; 2.7 uIU/ml is the average value), serum thyroglobulin (less than 40 ng/ml or more than 40 ng/ml, reference value is less than 40 ng/ml), tumor size (less than 1 cm, 1 cm-2 cm, more than 2 cm); 2)post-surgical data: extrathyroidal extension (no capsule contacting, invading capsule and violating surrounding tissues), multifocality (absent or present), bilateral tumor (absent or present), Hashimoto’s thyroiditis (absent or present), nodular goiter (absent or present) and CLNM (absent or present). All pathological results were obtained separately by two experienced pathologists; in case of discrepancy, further confirmation was made.

### Statistical Analysis

In this study, the SPSS 22.0 software was used for data analysis. Continuous measurement data were expressed as $\bar{\text{x}}$ ± S. Univariate logistic regression analysis or chi-square test was performed for various variables. Multivariate analysis was performed by multivariate logistic regression analysis. P<0.05 was considered statistically significant. The test level α was 0.05 on both sides. The process of establishing the nomogram used the R 386 3.5.2 software, using the “rms” software package to construct the nomogram. Through 1000 internal test plots, the calibration curve was compared with the predicted CLNM and actual CLNM rates to evaluate the accuracy of the nomogram. The area under the curve of the ROC curve was plotted according to the predicted CLNM probability to test the diagnostic efficiency.

## Results

### Baseline Characteristics and Pathological Features

Based on clinical features, the 907 subjects were divided as follows: age range of 17-89 years, with 421 younger than 45 years and 486 older than 45 years (average age was 45.6 ± 12.8); 235 males and 672 females, with a male to female ratio of 1:2.43; 428 (47.2%) cases had no CLNM and 479 (52.8%) had CLNM. The remaining features are shown in **Table 1**.

**Table 1:** Clinicopathological characteristics of the 907 PTC patients.

Parameter	<45 years (n = 421)		≥45 years (n = 486)	
	No.	Rate (%)	No.	Rate (%)
Gender				
Male	130	31.9	105	21.6
Female	291	68.1	381	78.4
Hashimoto’s thyroiditis				
Absent	296	70.3	376	77.3
Present	125	29.7	110	22.7
Tumor size (cm)				
≤1	170	40.6	213	43.8
1-2	180	42.8	192	39.6
>2	71	16.6	81	16.6
Multifocality				
Absent	277	65.8	318	65.4
Present	144	34.2	168	34.5
Bilateral tumor				
Absent	323	76.7	379	77.9
Present	98	23.3	107	22.1
ETE				
No capsule contacting	120	28.5	117	24.0
Invading capsule	251	59.6	305	62.9
Violating surrounding tissues	50	11.9	64	13.1
Duration of chief complaint				
≤1year	88	20.9	354	72.9
>1year	333	79.1	132	27.1
Margin in ultrasonography				
Clear	233	55.3	321	66.0
Unclear	188	44.7	165	34.0
Calcification in ultrasonography				
Absent	109	25.9	157	32.3
Present	312	74.1	329	67.7
Aspect ratio				
≤1	225	53.4	241	49.5
>1	196	46.6	245	50.5
TSH	2.7uIU/ml ± 5.2			
≤2.7 uIU/ml	319	75.8	325	66.8
>2.7 uIU/ml	102	24.2	161	33.2
Nodular goiter				
Absent	270	64.1	247	50.8
Present	151	35.9	239	49.2
Thyroglobulin				
≤40 ng/ml	350	83.1	379	77.9
>40 ng/ml	71	16.9	107	22.1
CLNM				
Present	270	64.1	209	43.1
Absent	151	35.9	277	56.9

PTC, papillary thyroid carcinoma; ETE, extra thyroidal extension; CLNM, **c**entral compartment lymph node metastasis.

### Single-Factor Analysis of CLNM

In the <45 years subgroup, 270 (64.1%) of all 421 patients had CLNM, versus 209 (43.0%) of 486 cases older than 45 years old, indicating a statistically significant difference (χ2 = 39.76, P<0.05). Univariate logistic regression analysis showed that in the overall population, gender, age, tumor size, bilateral tumor, degree of capsule invasion, margin on ultrasonogram, calcification on ultrasonogram and serum thyroglobulin had significant associations (P<0.05) with CLNM, and there were no significant associations of chief complaint duration, multifocality, tumor aspect ratio, TSH, Hashimoto’s thyroiditis and nodular goiter with CLNM (P>0.05). In the <45 years subgroup, gender, tumor size, multifocality, bilateral tumor, degree of capsule invasion, Hashimoto’s thyroiditis and serum thyroglobulin had significant associations (P<0.05) with CLNM, and there were no significant associations of chief complaint duration, margin on ultrasonogram, calcification on ultrasonogram, tumor aspect ratio, TSH and nodular goiter with CLNM (P>0.05). In the ≥45 years subgroup, gender, tumor size, multifocality, bilateral tumor, margin on ultrasonogram and calcification on ultrasonogram had significant associations (P<0.05) with CLNM. There were no significant differences in the associations of chief complaint duration, tumor aspect ratio, TSH and nodular goiter with CLNM (P>0.05) (**Table 2**).

**Table 2:** Predictive factors of CLNM in PTC patients according to univariate logistic analysis.

Parameter	<45 years (n = 421)			≥45 years (n = 486)			Total
	CLNM(+) (n = 270)	CLNM(-) (n = 151)	P	CLNM(+) (n = 209)	CLNM(-) (n = 277)	P	P
Gender			0.006			0.016	0.000
Male	96	34		56	49		
Female	174	117		153	228		
Tumor size (cm)			0.000			0.000	0.000
≤1	91	79		70	143		
1-2	120	60		89	103		
>2	59	12		50	31		
Multifocality			0.065			0.008	0.002
Absent	169	108		123	195		
Present	101	43		86	82		
Bilateral			0.051			0.002	0.000
Absent	199	124		149	230		
Present	71	27		60	47		
ETE			0.001			0.004	0.000
No capsule contacting	60	60		36	81		
Capsule invading	172	79		138	167		
Violation in surrounding tissues	38	12		35	29		
Duration of chief complaint			0.515			0.045	0.054
≤1 year	53	35		162	192		
>1 year	217	116		47	85		
Margin of ultrasonography			0.930			0.012	0.011
Clear	149	84		125	196		
Unclear	121	67		84	81		
Calcification in ultrasonography			0.068			0.015	0.001
Absent	62	47		55	102		
Present	208	104		154	175		
Aspect ratio			0.640			0.424	0.604
≤1	142	83		108	133		
>1	128	68		101	144		
Nodular goiter			0.085			0.684	0.588
Absent	165	105		104	143		
Present	105	46		105	134		
TSH			0.795			0.591	0.995
≤2.7 uIU/ml	209	110		137	188		
>2.7 uIU/ml	61	41		72	89		
Thyroglobulin			0.000			0.661	0.012
≤40 ng/ml	209	141		161	218		
>40 ng/ml	61	10		48	59		
Hashimoto’s thyroiditis			0.007			0.555	0.354
Absent	202	94		159	217		
Present	68	57		50	60		

### Multi-Factor Analysis of CLNM

Based on the above univariate analysis, in the overall population, factors that may have associations with CLNM (P<0.05), including gender, age, tumor size, multifocality, bilateral tumor, degree of capsule invasion, margin on ultrasonogram, calcification on ultrasonogram and serum thyroglobulin, were included in the multivariate logistic regression model. The results showed that large tumor size (P<0.05), extrathyroidal extension (P<0.05), male gender (OR=1.797, 95% CI 1.288-2.506; P<0.05), age <45 (OR=2.426, 95% CI 1.818–3.238; P<0.05) and calcification (OR=1.376, 95% CI 1.004–1.885; P<0.05) were independent risk factors for CLNM. In the <45 years subgroup, factors that may have associations with CLNM (P<0.05), including gender, tumor size, degree of capsule invasion, Hashimoto’s thyroiditis and thyroglobulin, were included in the multivariate logistic regression model. The results showed that large tumor size (P<0.05), extra-thyroid extension (P<0.05) and thyroglobulin level >40 ng/ml (OR=2.985, 95% CI 1.379-6.462; P<0.05) were independent risk factors for CLNM; meanwhile, Hashimoto’s thyroiditis (OR=0.532, 95% CI 0.324-0.874; P<0.05) were protective factors of CLNM. Gender showed no significant associations (P>0.05). In the ≥45 years subgroup, factors that may have associations with CLNM (P<0.05), including gender, tumor size, multifocality, bilateral tumor, margin on ultrasonogram, calcification on ultrasonogram, age, extrathyroidal extension and duration of chief complaint were included in the multivariate logistic regression model. The results showed that large tumor size (P<0.05), extra-thyroid extension (P<0.05), unclear margin under ultrasonography (OR=1.604, 95% CI 1.065-2.416; P<0.05), the male gender (OR=2.009, 95% CI 1.257-3.212; P<0.05) were independent risk factors for CLNM. Multifocality, bilateral tumor, calcification on ultrasonogram and duration of chief complaint had no significant associations in multivariate analysis (P>0.05) (**Table 3**).

**Table 3:** Predictive factors of CLNM in PTC patients in multiple logistic regression analysis.

Parameter	<45 years		≥45 years		Total
	p	Adjusted OR (95% CI)	p	Adjusted OR	P
Age	——	——	——	——	0.000
Gender	——	——	0.004	2.009 (1.257-3.212)	0.001
Tumor size (cm)	0.010		0.000		0.000
1-2	0.072	1.538 (0.963-2.457)	0.022	1.651 (1.077-2.531)	
>2	0.004	3.122 (1.451-6.719)	0.000	3.215 (1.818-5.683)	
ETE	0.003		0.018		0.000
Invading capsule	0.005	1.962 (1.222-3.151)	0.013	1.841 (1.140-2.973)	
Violating surrounding tissues	0.005	3.106 (1.416-6.811)	0.013	2.345 (1.198-4.590)	
Margin in ultrasonography	——	——	0.024	1.604 (1.065-2.216)	
Hashimoto’s thyroiditis	0.010	1.901 (1.167-3.097)	——	——	
Thyroglobulin	0.004	3.020 (1.418-6.432)	——	——	
Calcification	——	——	——	——	0.047
Constant	0.001	0.265	0.000	0.169	

### Nomogram Building and Analysis

**Figure 3:**
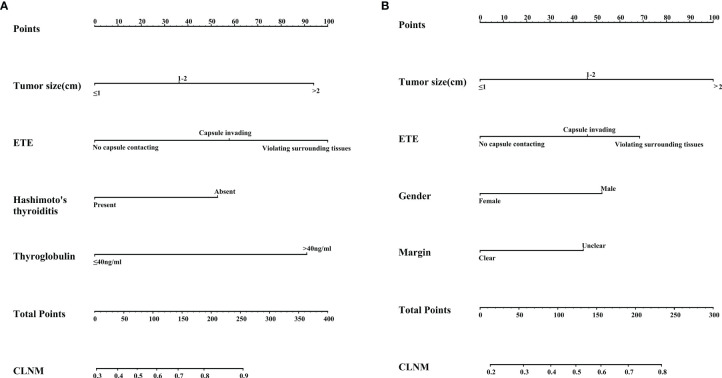
**(A)** Nomogram for predicting CLNM in the subgroup with age<45 years; **(B)** Nomogram for predicting CLNM in the subgroup with age ≥45 years.

Based on the above multivariate logistic regression analysis, a nomogram predictive model was created with the “rms” software package and analyzed with the R software, as shown in **Figure 3A, B**). Tumor size and extrathyroidal extension are important in CLNM in different age groups. In the <45 years subgroup, serum thyroglobulin and extrathyroidal extension accounted for a high weight of CLNM. At the same time, Hashimoto’s thyroiditis had a weak protective effect on CLNM. In the ≥45 years subgroup, unclear margin under ultrasonography and male gender were risk factors for CLNM.

**Figure 4:**
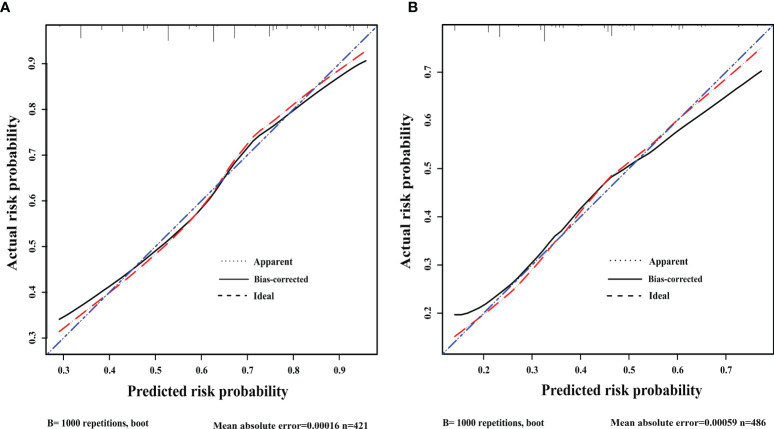
**(A)** Discrimination plot. The nomogram predictive model showed that in patients <45 years old, the average absolute error of the actual risk probability and the predicted risk probability of this model was 0.00016. **(B)** Discrimination plot. The nomogram predictive model showed that in ≥45 years, the average absolute error of the actual risk probability and the predicted risk probability of this model was 0.00059.

**Figure 5:**
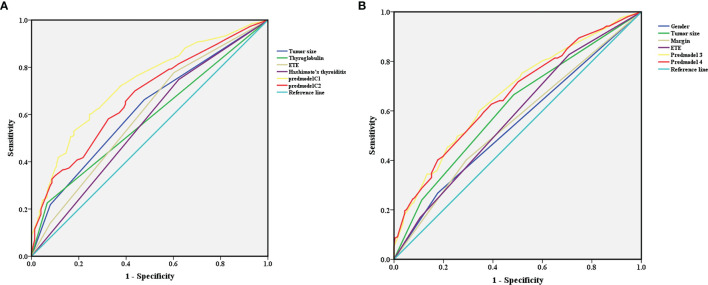
**(A)** In the subgroup with age <45 years, receiver operating characteristic analysis demonstrated an area under the curve(AUC) of 0.729 (95% CI 0.680-0.777; P<0.05). **(B)** In the subgroup with age ≥45 years, receiver operating characteristic analysis demonstrated an AUC of 0.668 (95% CI 0.619-0.716; P<0.05).

After 1000 internal verifications, an internal alignment curve was plotted. In the subgroup with age <45 years, the nomogram predictive model showed the average absolute error of the actual risk probability and the predicted risk probability of the model was 0.00016 (**Figure 4A**). The area under the ROC curve (AUC; **Figure 5A**, line predmodel 1) was 0.729 (95% CI 0.680-0.777; P<0.05), while the AUC based on the overall population was 0.675 (95% CI 0.623-0.727) (**Figure 5A**, line predmodel 2). In the subgroup with age ≥45 years, the average absolute error of the actual risk probability and the predicted risk probability of the model was 0.00059 (**Figure 4B**); the AUC (**Figure 5B**, line predmodel 3) was 0.668 (95% CI 0.619-0.716; P<0.05). The AUC based on the overall population was 0.661 (95% CI 0.612-0.710; P<0.05) (**Figure 5B**, line predmodel 4).

## Discussion

In the clinical treatment of thyroid papillary carcinoma, there are still many controversies in certain aspects (19, 20), including the scope of thyroid papillary gland resection and the choice between central and lateral neck prophylactic dissection. In the latest ATA guidelines (20), it was pointed out that in cases with metastasis lymph nodes before the operation, therapeutic central compartment lymph node dissection is recommended, but in cases without central compartment lymph node metastasis before the operation, preventive lymph node dissection is only conducted in progressive disease. The guideline was based on the survival time of patients, not postoperative recurrence, and some data (4) showed that preventive lymph node dissection can reduce the postoperative recurrence of thyroid papillary carcinoma. Because of the shielding of bone and trachea, the detection rate of central compartment lymph nodes by preoperative ultrasound is very low, with a sensitivity of less than 30% (21). Metastatic lymph nodes in PTC will have the characteristics of increased size, rounded shape, absence of a visible hilum, irregular reflection pattern, unsharp borders, cystic change, calcifications, and nonhilar vasculature (22, 23). However, if PTC is complicated with Hashimoto’s thyroiditis, we may obtain false-positive results. In our center, all patients diagnosed with thyroid papillary carcinoma underwent routine prophylactic central compartment lymph node dissection at least on one side. In all of patients, only 21 patients described the abnormal changes of central compartment lymph node at ultrasonography, of which 16 were proved to have CLNM. In the context of this debate, a more sensitive and accurate method for identifying preoperative central compartment lymph nodes becomes important.

Three systematic reviews (9, 11, 12) of papillary thyroid carcinoma showed gender, age, tumor size, multifocality, capsular invasion, lymphovascular invasion, tumor location, lymphocytic thyroiditis, bilateral tumor, extrathyroidal extension, lateral cervical lymph node metastasis, histological subtype of the tumor and BRAF gene mutations may be risk factors for central compartment lymph node metastasis. There are other studies on proteomics and fibronectin expression in CLNM (24, 25). In almost all studies (26–28), age, tumor size, gender, multifocality and extrathyroidal extension were considered risk factors for CLNM, but all these reports were based on the overall population without age stratification. It is well-known that age is an important factor affecting the biological behavior of the thyroid, and an important prognostic indicator in thyroid cancer (13). Differentiated thyroid carcinoma is the only tumor with age included in TNM staging. In the 8^th^ edition of AJCC (14), elderly patients are considered to have a higher death risk; however, PTC shows more aggressive biological behavior in younger groups (29, 30). Studies have shown that the types of gene mutations in PTC are different in distinct age stratification (31). The importance of age is also reflected in other risk predictive models, including AMES, DAMES and MACIS. In this study, we found that the proportion of CLNM was higher in the younger group, and the proportion of males (P<0.05) and Hashimoto’s thyroiditis incidence (P<0.05) in younger patients were higher than in older patients.

Based on different age groups, we found that papillary thyroid cancer has different metastatic patterns. In the multi-factor study, it was found that in the subgroup with age <45 years, tumor size, extrathyroidal extension, Hashimoto’s thyroiditis and serum thyroglobulin levels were independent risk factors for CLNM. However, in the subgroup with age ≥45 years, tumor size, extrathyroidal extension, unclear margin under ultrasonography and male gender were independent risk factors for CLNM. Previous studies based on the overall population without age stratification also found that in different studies, factors affecting CLNM are different, which may be caused by the uneven distribution of the population. In this study, as in the overall population, tumor size, extrathyroidal extension, male gender, younger age and calcification were independent risk factors for CLNM. In the present study, we divided the overall population into different subgroups according to age, and the predictive model had a higher area under the ROC curve than the model constructed according to the overall population. Whether this difference is caused by different tumor subtypes or the body’s immune status remains to be further studied (15–17). This would enable more accurate clinical decisions in patients of different ages.

In addition, we found that thyroglobulin level was an important factor associated with CLNM in the subgroup with age <45 years. In previous studies (32, 33), serum thyroglobulin could generally be assessed by lymph node puncture before surgery to determine the status of lymph node metastasis or tumor recurrence after surgery. Besides, previous studies (27, 28) have shown that male patients are more likely to have CLNM in PTC, which was reflected in patients older than 45 years, but this trend was not obvious in younger patients. It is well known that differences in sex hormones are more common in younger patients, so the above finding is intriguing. It is worth mentioning that in the younger group, Hashimoto’s thyroiditis may be a protective factor of CLNM, but not in older cases. Hashimoto’s thyroiditis represents an autoimmune inflammation of the thyroid gland that is common in young patients. Studies have shown that Hashimoto’s thyroiditis is a stimulating factor in the occurrence of thyroid papillary carcinoma through immune activation. There are similar findings in existing studies (34–36), with no indication of the role of age.

Our research had limitations as a single-center study with no external validation. Further multi-center studies are required for verifying these findings. In addition, due to the limited number of patients, low AUCs were obtained, and more data are needed to make our results more stable. In addition, the focus of our research is to predict whether there is CLNM, but it cannot be used to predict the number and size of lymph node metastasis, for which we need do further research. Furthermore, long-term follow-up is needed to compare the postoperative recurrence risk of pN1 patients in different age groups, in order to further clarify the impact of age on tumor metastasis and recurrence.

## Summary

Central compartment lymph nodes of papillary thyroid carcinoma in different age groups may have distinct patterns. The effects of extrathyroidal extension and tumor size in CLNM are reflected in all age groups. In young patients, preoperative serum thyroglobulin levels and the presence of Hashimoto’s thyroiditis may affect CLNM, while gender is not significantly important. In older cases, males were more likely to have CLNM. These differences may be explained by different hormone levels and immune states in patients of different ages, which may inform decision-making for individual patients in the clinic.

## Data Availability Statement

The raw data supporting the conclusions of this article will be made available by the authors, without undue reservation.

## Author Contributions

CY, XH, ZC, and YW contributed to the conception and design of the study. CY, ZC, and YW organized the database. CY performed the statistical analyses, wrote the first draft of the manuscript, and wrote sections of the manuscript. All authors contributed to manuscript revision and read and approved the submitted version. All authors contributed to the article and approved the submitted version.

## Funding

This work was partially supported by the National Natural Science Foundation of China (81672641).

## Conflict of Interest

The authors declare that the research was conducted in the absence of any commercial or financial relationships that could be construed as a potential conflict of interest.

## Publisher’s Note

All claims expressed in this article are solely those of the authors and do not necessarily represent those of their affiliated organizations, or those of the publisher, the editors and the reviewers. Any product that may be evaluated in this article, or claim that may be made by its manufacturer, is not guaranteed or endorsed by the publisher.
